# Green synthesis and characterization of Bi₂O₃ nanoparticles using *Cuminum cyminum* seed for antimicrobial and cytotoxic evaluation

**DOI:** 10.1186/s11671-026-05046-y

**Published:** 2026-10-20

**Authors:** Muhammad Gohar, Atika Mumtaz, Umar Asim, Kanwal Shahzadi, Sidra Majeed, Muhammad Ismail, Elias Assayahegn, Shabnam Javed

**Affiliations:** 1 Department of Chemistry, University of Southern Punjab, Multan, 60750 Punjab Pakistan; 2 Institute of Chemistry, University of the Punjab, Lahore, Pakistan; 3 Energy Lab, Green Technologies and Advanced Matter (GREAT) Research Group, Faculty of Science and Technology, Universiti Malaysia Sabah, Kota Kinabalu, Sabah, 88400 Malaysia; 4 Department of Chemistry, CNCS, Mekelle University, P.O.Box 231, Mekelle City, Tigray, Ethiopia

**Keywords:** Bismuth oxide nanoparticles, Bi_2_O_3_, Green synthesis, *Cuminum cyminum*, Plant extract, Structural characterization, Antimicrobial activity, Antifungal activity, Cytotoxicity, Cancer cell lines

## Abstract

Green synthesis provides an alternative to traditional chemical processes of nanomaterials preparation, which is cost-effective and environmentally benign. This paper has demonstrated synthesis of bismuth oxide nanoparticles (Bi_2_O_3_ NPs) using *Cuminum cyminum* seed extract as a natural reducing and stabilizing agent of bismuth nitrate precursor. The formation of nanoparticles was first signaled by a change of color of reaction mixture to be visible and subsequent analysis by physicochemical characterization. The UV-Visible spectroscopy displayed absorbance at 300 nm, and FTIR gave results that showed Bi-O-related absorption and phytochemical functional groups, which indicated the formation of bismuth oxide nanoparticles stabilized by plant-derived biomolecules. The crystalline character of the nanoparticles produced was verified by X-ray diffraction analysis with estimated crystallite size of approximately 136.9 nm. SEM analysis revealed irregular and strongly agglomerated structures. Image analysis of clearly distinguishable agglomerates indicated apparent dimensions of approximately 0.20–2.00 μm, with highest frequency in the 0.60–0.80 μm interval; however, individual primary particles could not be resolved reliably. The Bi₂O_3_ nanoparticles biosynthesized had antimicrobial and antifungal effects against *Escherichia coli*, *Staphylococcus aureus*, *Candida albicans*, and *Aspergillus niger* with *Candida albicans* being the most sensitive. Furthermore, cytotoxicity studies revealed that HeLa and MCF-7 cell proliferation were greatly inhibited by synthesized nanoparticles, which indicates the possibility of biomedical applications of the synthesized nanoparticles. All in all, this work shows an easy and environmentally friendly path to green synthesis of Bi_2_O_3_ nanoparticles using *Cuminum cyminum* seed extract and reflects on their potential to have antimicrobial and cytotoxic capabilities.

**Graphical abstract:**

**Figure:**
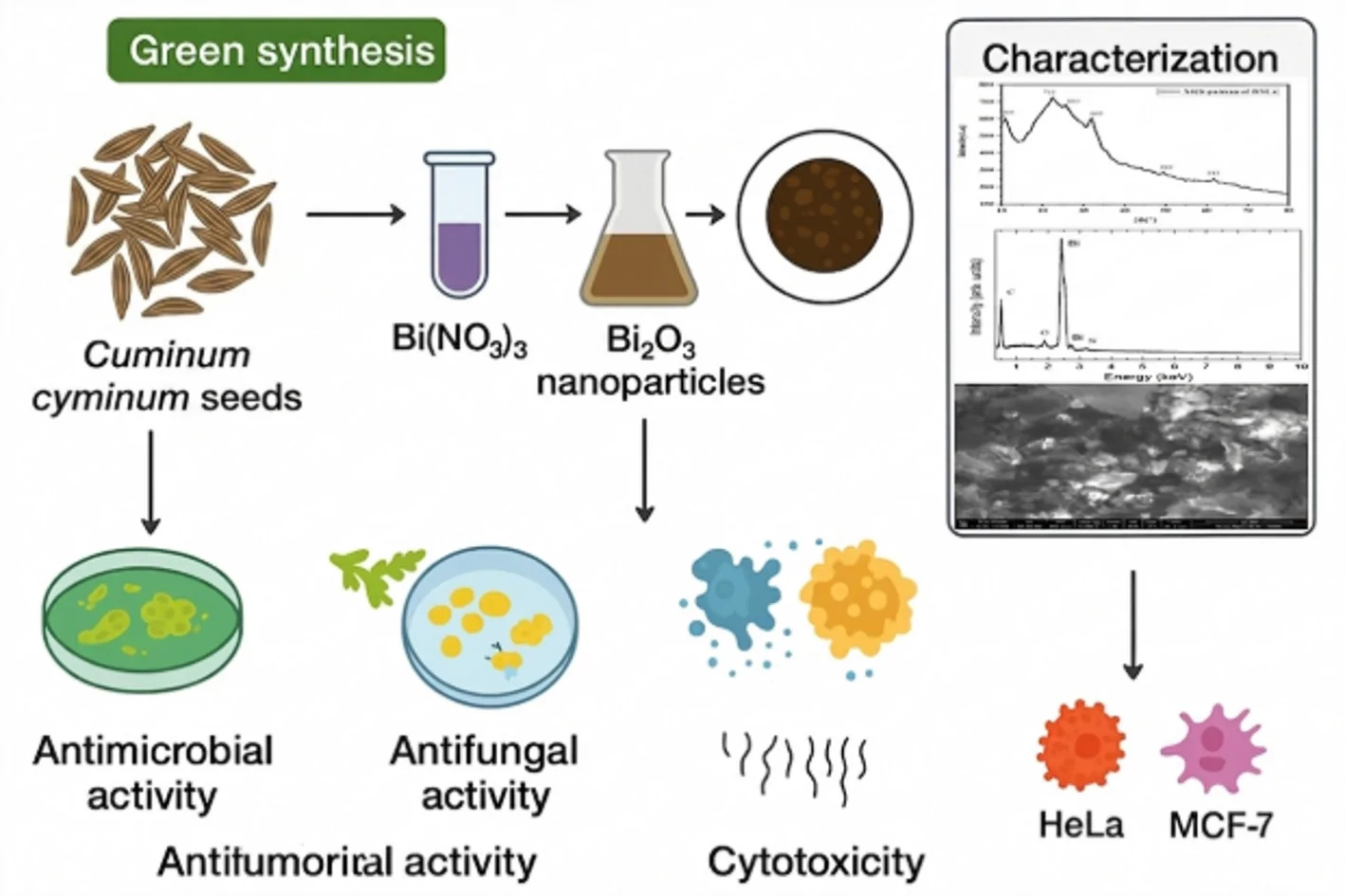

## Introduction

Nanotechnology has helped to improve biomedical and environmental studies through the design and use of nanomaterials that have unique physicochemical and biological properties. Of these, metal and metal oxide nanoparticles have received a lot of attention because of their size that can be adjusted, high surface-area-to-volume ratio, and wide range of applications [1]. Over the last few years, more and more focus has been given to sustainable and eco-friendly synthesis methods. Plant-mediated green synthesis has been identified as an alternative in this regard, as a more favorable means of synthesis compared to standard chemical and physical techniques. This method takes advantage of naturally occurring biomolecules like alkaloids, flavonoids, terpenoids, proteins, and phenolic compounds as reducing and stabilizing agents, thus removing the necessity of using hazardous chemicals and harsh reaction conditions and providing a simple, inexpensive, and environmentally friendly method of nanoparticle synthesis [2, 3].

Bismuth-based nanoparticles, especially bismuth oxide nanoparticles (Bi_2_O_3_ NPs), have become a topic of growing interest among other nanomaterials because of their distinctive structural and functional properties [4]. Bismuth is a post-transition metal characterized with relatively low toxicity, high atomic number, and high X-ray attenuation characteristics [5], which makes it a good choice to be used in imaging, catalysis, and biomedical research. Moreover, bismuth oxide nanoparticles have been shown to possess good potential against bacteria, fungi, and cancer cells, indicating their potential in biomedical and environmental applications [6]. Relative to the widely researched nanoparticles like silver and gold, the bismuth-based materials [7] have been found to be more biocompatible and less likely to cause undesirable cytotoxic responses, therefore, they are good candidates in biological applications. In spite of these benefits, bismuth oxide nanoparticles synthesized by plants is relatively little studied meaning that it requires further elaboration.

Nanoparticles green-synthesized are considerably affected by the nature of the plant extract employed since the phytochemical composition determines the nucleation, growth and stabilization mechanisms. The medicinal plant *Cuminum cyminum* (cumin) is a rich source of bioactive compounds including phenolics, flavonoids and terpenoids including cuminaldehyde, limonene and cymene, which are antioxidant and antimicrobial [8, 9]. The phytochemicals have the potential to be significant in the nanoparticle fabrication as they may assist in converting precursors and also serve as surface-stabilizing agents, which may impact nanoparticle shape, dispersion, and biologic functionality [10].

Bismuth oxide nanoparticles are less studied compared to other green-synthesized nanomaterials even though they have a good combination of functionality and possible biocompatibility. As an example, silver nanoparticles are well-known due to the high antimicrobial activity, but can have a problem with cytotoxicity and microbial resistance. Gold nanoparticles have great biocompatibility and are however costly to use at large scales, whereas zinc oxide nanoparticles can produce reactive oxygen species that can cause oxidative stress in biology. Herein, Bi_2_O_3_ nanoparticles provide a potentially beneficial balance of biological activity, stability, and environmental compatibility, which makes them promising in terms of sustainable nanotechnology applications [11].

Recent reports have shown that bismuth-based nanomaterials synthesized through plant mediation can be produced to give significant biological characteristics on nanoparticles. As an example, Mahiuddin et al. (2022) found that plant extracts were used to greenly synthesize bismuth-based nanoparticles that had high antibacterial and antioxidant properties, whereas Shanthi et al. (2021) found plant-mediated bismuth nanoparticles to have cytotoxic effects on cancer cell lines. These results imply that plant biomolecules can also play a role in forming nanoparticles and can also increase their biological functions by interacting with surfaces [12, 13].

Phytochemicals found in the plant extracts might be involved in the process of hydrolysis [14], nucleation and stabilization in the presence of controlled conditions during green synthesis. Within alkaline and hot conditions, e.g. the conditions used in this research, it is easier to form bismuth oxide nanoparticles compared to metallic bismuth. Moreover, the biomolecules (of plant origin) may adsorb onto the surface of the nanoparticles and serve as capping agents to control the growth of the particles, to inhibit agglomeration, and to enhance their colloidal stability [15].

Surface-bound phytochemicals can also tend to alter the biological activity of green-synthesized nanoparticles, and can promote their contact with microbial and cellular systems. Past research has demonstrated that nanoparticles that are delivered by plants may be more antimicrobial active than the plant extracts of the same [16], which is probably because of augmented surface reactivity and enhanced bioavailability. Thus, nanoparticles obtained with the help of *Cuminum cyminum* extract can exhibit better biological effects because of the joint impact of the inorganic core and phytochemical surface functionalization [17].

Bismuth-based nanomaterials have also been studied (in addition to their use as antimicrobials) in more general biomedical applications because they have good physicochemical characteristics. Their biocompatibility and high atomic number make them appealing to imaging related applications, whereas their biological activity promises them to find applications in therapeutic research. Nevertheless, the current research is narrowed down to the green synthesis, characteristics, antimicrobial, antifungal, and cytotoxic of Bi_2_O_3_ nanoparticles [7, 18].

On the whole, the literature indicates that the synthesis of bismuth oxide nanoparticles through the mediation of plants is a promising, but under-researched field of study. Specifically, there are not a lot of systematic studies on the use of *Cuminum cyminum* [19] as a bioresource to produce Bi_2_O_3_ nanoparticle [20]. This demonstrates a gap in current research on comprehending the role of cumin-derived phytochemicals in the formation of nanoparticle and its effect on biological activity.

The purpose of this research is to create an eco-friendly and sustainable system of bismuth oxide nanoparticles (Bi_2_O_3_ NPs) production based on *Cuminum cyminum* seed extract, as well as to assess their physicochemical properties and biological functionality. The synthesized nanoparticles were determined under UV-Visible spectroscopy, FTIR, XRD and SEM methods and their antimicrobial, antifungal and cytotoxic qualities were examined. This paper is an addition to the emerging area of green nanotechnology in its quest to investigate the use of *Cuminum cyminum* as natural resource in preparation of biologically active Bi_2_O_3_ nanoparticles.

## Materials and methods

### Materials

Analytical grade reagents bismuth nitrate pentahydrate (Bi(NO₃)₃·5 H₂O, ≥ 98% purity) and sodium hydroxide (NaOH, ≥ 99% purity) were procured from Merck (Germany). *Cuminum cyminum* seeds were obtained from a local herbal supplier. Distilled deionised water was used throughout all preparative and washing steps. All glassware (beakers, conical flasks, centrifuge tubes) and equipment (centrifuge, pH meter, sonicator, water bath) were cleaned and rinsed with distilled water before use.

### Plant extract preparation

#### Cumin seed extract

**Fig. 1:**
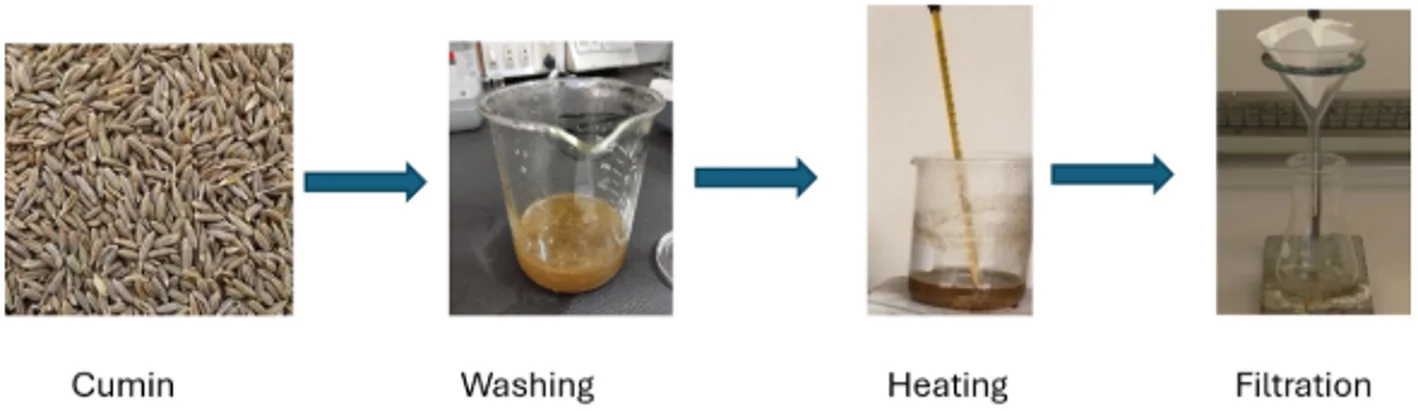
Preparation of cumin seed extract in distilled water for use as a natural reducing and stabilizing agent in the green synthesis of bismuth oxide nanoparticles

The Cuminum cyminum seeds that were used in this research were bought in a local herbal market in Lahore, Pakistan (31.5204°N, 74.3587°E). Cleaned Cuminum cyminum seeds were shade-dried and ground into a fine powder. Ten grams of this powder were mixed with 100 mL of distilled water and heated in a water bath at 50 °C for 20 min with occasional stirring. The mixture was filtered using Whatman No. 1 filter paper, and the filtrate was retained and stored at 4 °C [21, 22] (Fig. 1).

### Green synthesis of bismuth oxide nanoparticles (Bi₂O₃ NPs)

**Fig. 2:**
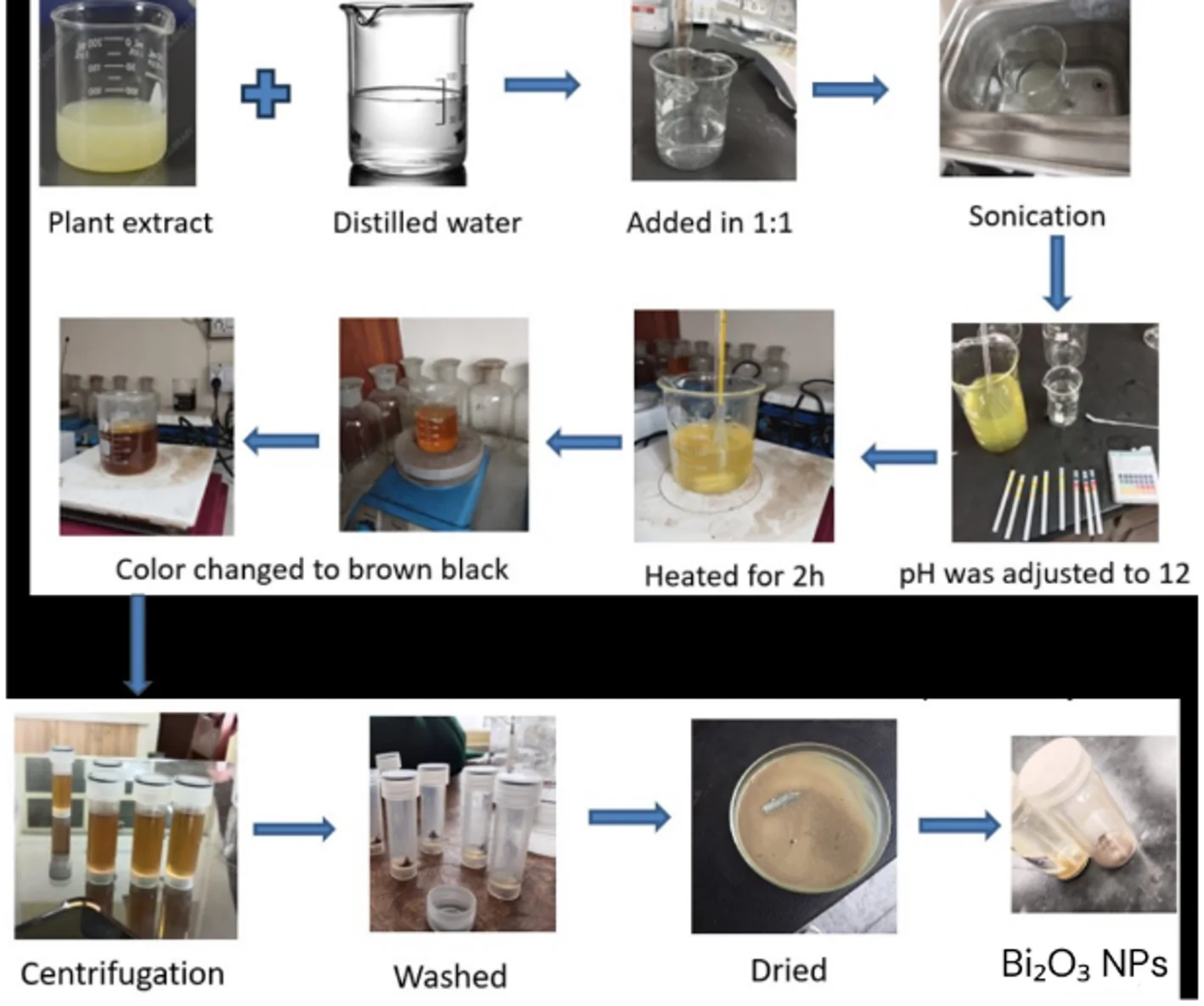
Synthesis of bismuth oxide nanoparticles (Bi_2_O_3_ NPs) using cumin seed extract as a natural reducing and stabilizing agent

For each synthesis of Bi₂O₃ NPs, 121.3 mg (0.25 mmol) of Bi(NO₃)₃·5 H₂O was dissolved in 25.0 mL of plant extract. Sonication of the solution was done to facilitate homogenization (20 min). The pH was adjusted to 12.3–12.4 by adding 3.5–4.0 mL of 4 M NaOH. This high pH was chosen to favor the hydrolysis of the Bi^3+^ ions and facilitate the formation of bismuth hydroxide/oxyhydroxide intermediacy species which can further be transformed into bismuth oxide nanoparticles under heating [23, 24]. Deprotonation of phytochemical functional groups, like a hydroxyl and carboxyl groups, contained in the plant extract also is preferred by the alkaline medium, which in turn enhances their reaction with bismuth species, as well as helps stabilize nanoparticles. To prepare reaction mixtures, a 2-hour incubation of the reaction mixtures at 80 °C in thermostatic water bath with constant stirring was conducted until a visible color change of yellow to deep red and ultimately to dark brown was observed, indicating the formation of nanoparticles. The solution was allowed to cool to a room temperature, centrifuged at 8,000 rpm and 10 min to harvest the nanoparticles, and washed thrice with distilled water, and dried in a vacuum oven at 65 °C 12 h (Fig. 2).

### Optimization of synthesis conditions

The effects of reaction pH, temperature, reaction time and cumin-extract volume on Bi₂O₃ formation were investigated using a one-factor-at-a-time approach. The tested pH values were 10, 11 and 12.3; reaction temperatures were 60, 80 and 100 °C; reaction times were 1, 2 and 3 h; and extract volumes were 15, 25 and 35 mL. During evaluation of each parameter, the remaining synthesis conditions were maintained constant. The resulting products were compared based on isolated yield, UV–Visible response and XRD phase matching. The selected synthesis conditions were those that provided the most reproducible product yield and strongest agreement with the identified Bi₂O₃ diffraction pattern.

### Characterization techniques

UV Visible spectroscopy was used to monitor the optical properties of nanoparticles between the 200–800 nm ranges, and spectra were taken at fixed times during the synthesis of the particle to record the development of the characteristic absorption features. The presence of functional groups and the presence of phytochemical capping agents in dried Bi_2_O_3_ NPs powder were identified using Fourier-transform infrared (FTIR) spectroscopy at dried Bi_2_O_3_ NPs powder in KBr mode or ATR mode and measured between 4000 –400 cm^-1^ to detect changes in O-H, C = O and BIO stretching bands. X-ray diffraction (XRD) with a Cu Kα radiation (λ = 1.5406 Å) was used to determine the crystalline phase and mean crystallite size with a scan of 20°–80°at 2θ (0.02°) and an estimate of the crystallite size was made by the Scherrer equation based on the highest intensity rhombohedral bismuth peaks. The surface morphology and aggregation behaviour of the biosynthesized sample of Bi₂O₃ were analysed by scanning electron microscope (SEM). The micrographs were taken on the SEM and the scale bar was used to size the images in order to calculate the SEM. The lateral dimensions of the clearly defined agglomerates were measured on the micrographs using the image-analysis software, and the apparent maximum lateral dimensions were used. Measurements were not made on severely overlapping structures and domains with indistinct boundaries. The measured dimensions were divided into intervals of 0.20 microns to draw the frequency-distribution histogram and a fitting curve to show the general trend of the distribution. The measurements were thus assumed to represent the apparent dimensions of the SEM-visible agglomerates, since the SEM images displayed highly agglomerated structures, which could not be clearly distinguished.

To determine the antimicrobial and antifungal activity, *Escherichia coli* (Gram-negative), *Staphylococcus aureus* (Gram-positive), *Candida albicans* and *Aspergillus niger* were used in the biological evaluation. The lowest concentration at which antibiotics can inhibit microorganisms was also determined by the broth microdilution technique, though zone of inhibition tests on agar plates and biofilm disruption experiments performed by crystal violet staining and optical density were also some helpful insights into microbial inhibition. The MTT assay was used to determine cytotoxicity of HeLa and MCF-7 human cancer cell lines subjected to different concentrations of Bi_2_O_3_ NPs and viability of cells were measured at 570 nm as compared to control sample cultures. The cell lines HeLa and MCF-7 were obtained from the department of biotechnology, University of the Punjab, Lahore, Pakistan, and then were cultivated under the standard laboratory conditions before using them in the experiments. Intracellular generation of reactive oxygen species (ROS) was also measured by the DCFH-DA probe when necessary, and a fluorescence reading of 485/530 nm was recorded to determine the oxidative stress caused by the exposure of Bi_2_O_3_ NPs.

### Statistical analysis

All antimicrobial, antifungal and cytotoxicity experiments were performed in triplicate, and the results are expressed as mean ± standard deviation. Antimicrobial and antifungal inhibition-zone data were analysed using two-way analysis of variance, followed by Tukey’s post-hoc test. Cytotoxicity data were analysed using two-way analysis of variance to evaluate the effects of nanoparticle concentration and exposure time. Statistical significance was accepted at *p* < 0.05.

## Results and discussion

### Green synthesis of bismuth oxide nanoparticles (Bi_2_O_3_ NPs)

This study employed cumin seed extract as a green reducing and stabilizing agent to synthesize bismuth oxide nanoparticles (Bi₂O₃ NPs) using a bismuth nitrate [Bi(NO_3_)_3_] solution. The cumin extract was combined with the bismuth nitrate solution and the mixture was subjected to controlled heating whereby a clear shift of color was witnessed as the yellow color changed to dark brown. This change of color is evidence of reduction of Bi^3+^ ions and the creation of Bi₂O₃ NPs. The emergence of dark brown color is an intrinsic property of metallic nanoparticle formation and it proves the effective production of Bi₂O₃ NPs in the reaction mixture. The above-mentioned findings are congruent with the earlier investigations on the green synthesis of metallic nanoparticles that proved the efficiency of cumin extract in facilitating the process of nanoparticle formation and stabilization.

### Characterization of Bi_2_O_3_ NPs

#### UV-visible analysis

**Fig. 3:**
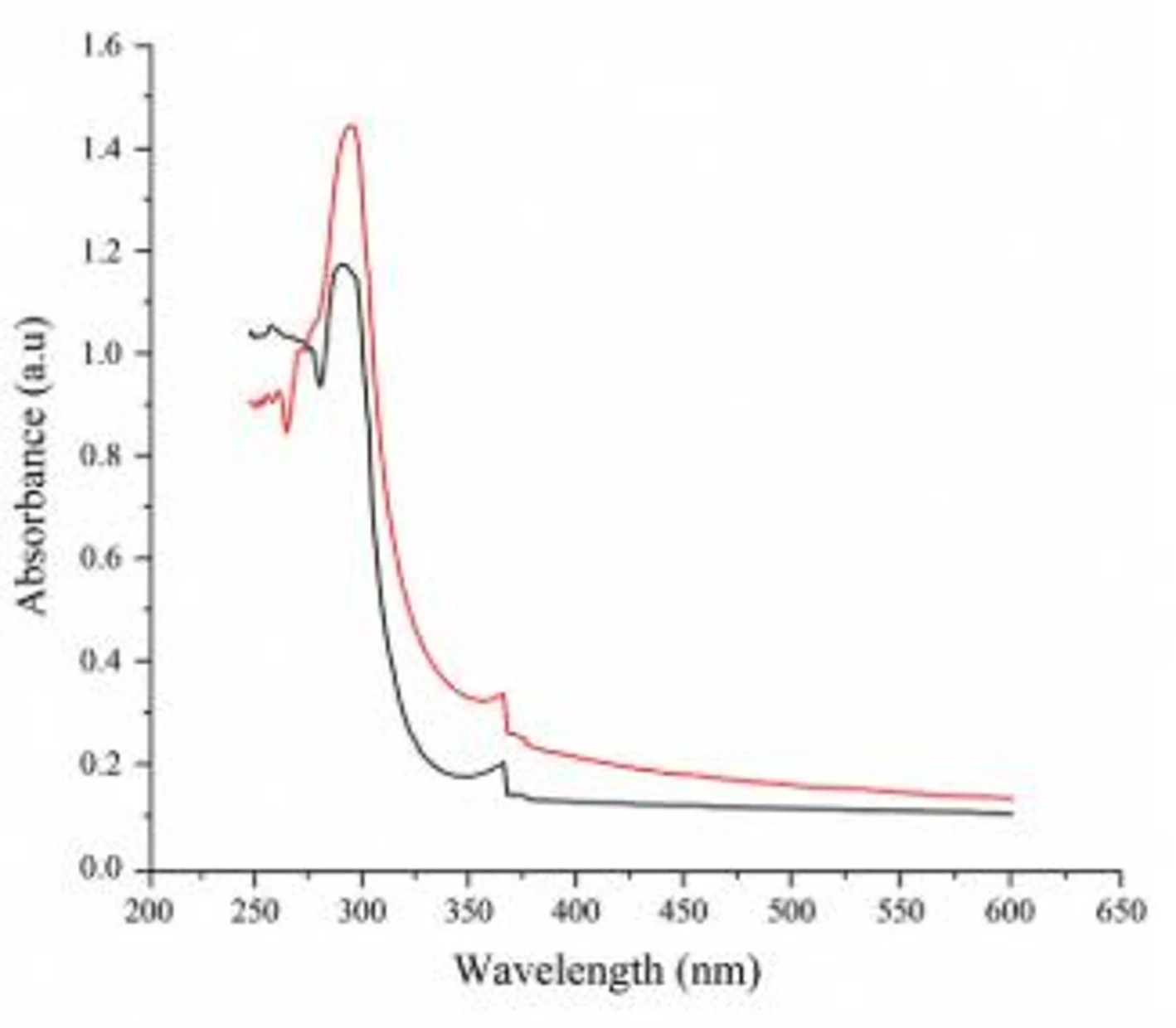
UV–Visible spectra of the Cuminum cyminum seed extract and biosynthesized Bi₂O₃ nanoparticle dispersion recorded over 200–800 nm under identical experimental conditions

Figure 3 compares the UV–Visible spectra of the *Cuminum cyminum* seed extract and the biosynthesized Bi₂O₃ nanoparticle dispersion. The cumin extract exhibited substantially higher and broader absorbance in the ultraviolet region, which can be attributed to the combined absorption of phenolic, flavonoid and other plant-derived compounds. In comparison, the Bi₂O₃ nanoparticle dispersion showed lower absorbance and a comparatively smooth decrease in intensity with increasing wavelength. The difference in spectral shape and intensity indicates modification of the optical response following interaction of the bismuth precursor with the phytochemical constituents of the extract.

No clearly resolved pair of corresponding absorption maxima was visible in the two spectra; therefore, a definite redshift or blueshift could not be established from the present measurements. Similarly, the spectrum did not show a distinct band that could be assigned confidently to localized surface plasmon resonance. Because Bi₂O₃ is a semiconductor oxide, the observed ultraviolet response is more cautiously attributed to electronic transitions involving the Bi–O system, together with possible contributions from surface-bound phytochemicals.

The relatively high absorbance of the cumin-extract spectrum may indicate excessive sample concentration and overlapping absorption from multiple phytochemical components. In nanoparticle dispersions, light scattering caused by particle agglomeration may also contribute to the measured optical intensity. Consequently, the UV–Visible results are interpreted qualitatively, while confirmation of Bi₂O₃ formation and crystalline phase is based principally on complementary FTIR and XRD analyses.

#### FTIR analysis

**Fig. 4:**
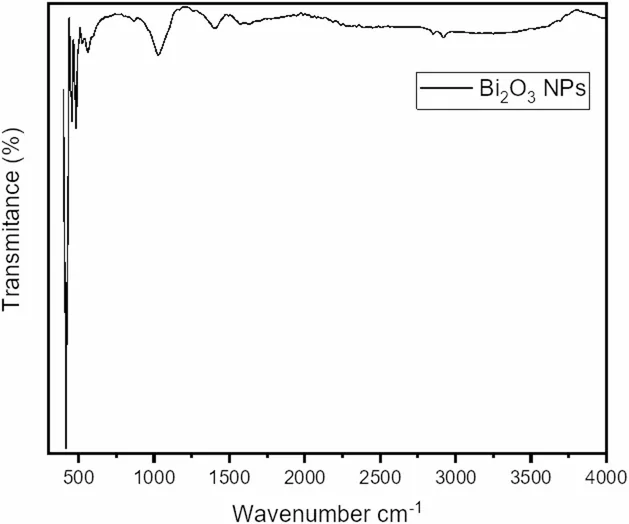
FTIR spectrum of the washed and dried Bi₂O₃ nanoparticle powder synthesized using *Cuminum cyminum* seed extract

Figure 4 shows the FTIR spectrum of the washed and dried Bi₂O₃ nanoparticle powder synthesized using *Cuminum cyminum* seed extract. The spectrum exhibits distinct absorption bands at 3698.20, 2920.52, 2851.34, 2018.35, 1990.58, 1962.33, 1588.05, 1396.18, 1259.36, 1139.05, 1096.52, 1017.38, 956.55, 855.65, 665.70, and 529.21 cm⁻¹. The peak at 3698.20 cm⁻¹ corresponds to O–H stretching vibrations of phenolic compounds or alcohols, indicating the presence of hydroxyl groups in cumin extract. The bands at 2920.52 cm⁻¹ and 2851.34 cm⁻¹ are attributed to C–H stretching vibrations of aliphatic hydrocarbons and fatty acids. C = C stretching in aromatic rings is attributed to the peak at 1588.05 cm^− 1^ which indicates the presence of phenolic or flavonoid products, whereas C-H bending vibration is attributed to the band at 1396.18 cm^− 1^ which indicates the presence of methyl or methylene groups [25, 26]. The absorption at 1259.36 cm^− 1^ relates to C-O vibrations in ethers, esters, or alcohols and the additional peaks at 1139.05 cm^− 1^ and 1096.52 cm^− 1^ are other indications of C-O vibrations in alcohols, esters or phenols. The 1017.38 cm^− 1^ band could be associated with C-N or C-O vibration, and the peak at 956.55 cm^− 1^ is connected with C-H bending in cyclic or aromatic structures. These two peaks of 855.65 cm^− 1^ and 665.70 cm^− 1^ represent the C-H out-of-plane bending vibrations, which are common to substituted aromatic compounds. Bending modes in inorganic structures are normally related to a peak that is lower than 529.21 cm^− 1^ and this may indicate the interaction of the bismuth ions and organic molecules in the extract. It indicates the presence of these low-frequency peaks, which implies the successful formation of the Bi₂O₃ NPs with Bi-O and Bi-O-Bi bonds stabilized by phytochemicals. Flavonoids, alkaloids and phenols are organic compounds that can be both used to reduce bismuth ions, and to maintain the nanoparticles in aqueous media. All in all, FT-IR analysis proves the presence of functional groups of cumin extract on the surface of nanoparticles and justifies the successful production of structurally stable bismuth oxide nanoparticles that can be used in further bio- and environmental-related research [27].

#### XRD analysis

**Fig. 5:**
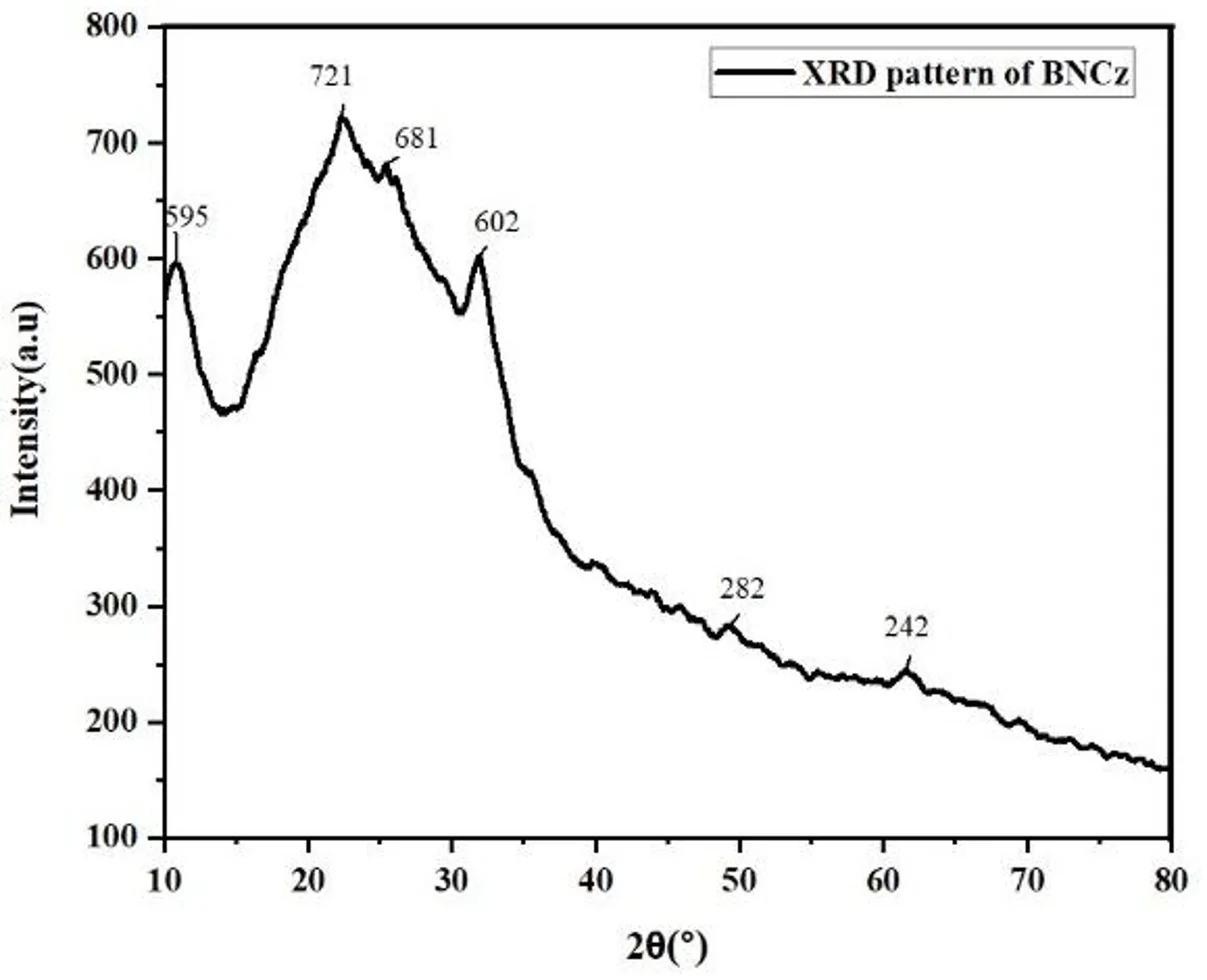
XRD pattern of the biosynthesized bismuth oxide material showing reflections tentatively assigned to monoclinic α-Bi₂O₃ (JCPDS/PDF No. 41-1449) and tetragonal β-Bi₂O₃ (JCPDS/PDF No. 27–0050). The unmatched low-angle reflections near 11.0° and 23.5° are marked with asterisks

The XRD pattern of the synthesized material is presented in Fig. 5. Diffraction reflections were observed at 2θ values of approximately 11.0°, 23.5°, 25.3°, 31.9°, 48.9° and 62.1°. Comparison with standard diffraction patterns indicated partial matching with monoclinic α-Bi₂O₃ and tetragonal β-Bi₂O₃ (Table 1). The reflections at approximately 48.9° and 62.1° were assigned tentatively to the (104) and (232) planes of monoclinic α-Bi₂O₃, respectively, in agreement with JCPDS/PDF No. 41-1449. The reflection at approximately 31.9° was assigned to the (002) plane of tetragonal β-Bi₂O₃, consistent with JCPDS/PDF No. 27–0050. The reflection near 25.3° occurs in a region where reflections from α- and β-Bi₂O₃ may overlap therefore, it was not used as evidence for a single polymorphic phase. Table 1

**Table 1:** XRD peak indexing and phase assignment of the biosynthesized Bi₂O₃ sample

Experimental 2θ (°)	Closest standard 2θ (°)	Tentative hkl	Tentative phase	Interpretation
11.0	—	—	Unidentified	Low-angle reflection not matched to the principal α- or β-Bi₂O₃ peaks
23.5	—	—	Unidentified	Possible minor precursor/secondary-phase contribution; no definitive assignment
25.3	25.6–25.7	(102)/(210)	α- or β-Bi₂O₃	Overlapping region; phase assignment is not unique
31.9	31.7	(002)	β-Bi₂O₃	Consistent with tetragonal β-Bi₂O₃
48.9	48.5	(104)	α-Bi₂O₃	Consistent with monoclinic α-Bi₂O₃
62.1	62.3	(232)	α-Bi₂O₃	Consistent with monoclinic α-Bi₂O₃

The standard α-Bi₂O₃ pattern includes reflections at approximately 25.6°, 48.5° and 62.3°, while β-Bi₂O₃ includes a reflection at approximately 31.7.

A low-angle reflection was observed at approximately 2θ = 11.0°. This feature did not correspond to the principal diffraction reflections of either α-Bi₂O₃ or β-Bi₂O₃. Similarly, the reflection at approximately 23.5° could not be assigned confidently to either Bi₂O₃ polymorph. These low-angle features may originate from a residual precursor, plant-derived surface material, a minor secondary phase or a sample-holder/background contribution. Because no definitive database match was obtained, both features were reported as unidentified reflections and were excluded from crystallite size calculations.

Overall the XRD pattern suggests the presence of crystalline Bi₂O₃ with possible coexistence of α- and β-phase contributions. However, because several reflections remained unmatched the pattern does not demonstrate phase pure Bi₂O₃. Additional high resolution XRD or complementary structural analysis would be required to identify the minor unidentified reflections conclusively.

#### SEM analysis

**Fig. 6:**
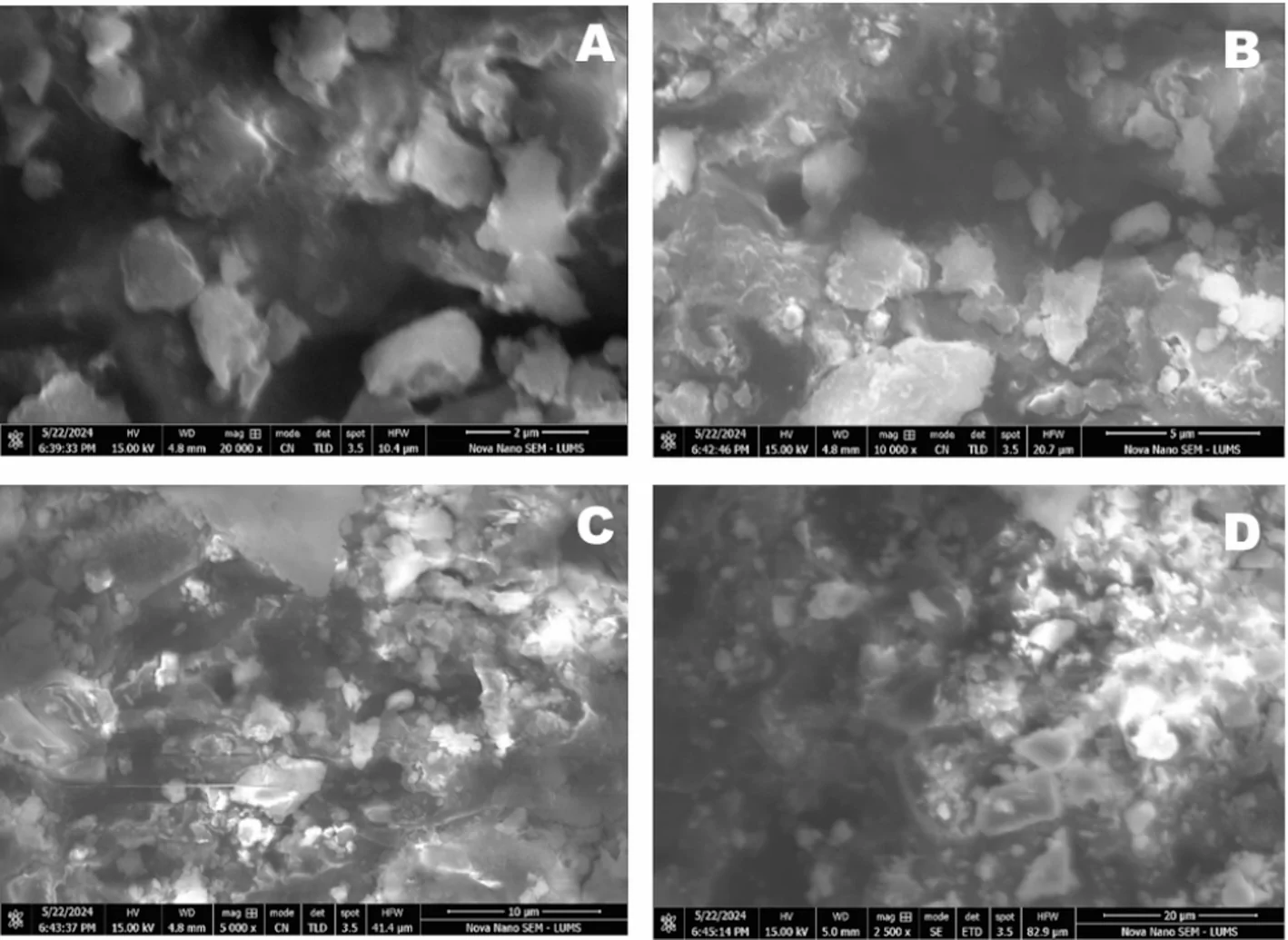
SEM micrographs of the biosynthesized Bi₂O₃ sample recorded at different magnifications: (**A**) 20,000× with a 2 μm scale bar, (**B**) 10,000× with a 5 μm scale bar, (**C**) 5,000× with a 10 μm scale bar and (**D**) 2,000× with a 20 μm scale bar. The images show irregular, rough and strongly agglomerated structures rather than clearly resolved individual primary nanoparticles

The SEM micrographs of the biosynthesized Bi₂O₃ sample are presented in Fig. 6A–D. The images show irregular, rough and strongly agglomerated structures. At higher magnification, the material appears to consist of densely packed domains in which the boundaries of individual primary particles cannot be distinguished reliably. At lower magnifications, larger heterogeneous aggregates with submicrometre-to-micrometre dimensions are visible.

The SEM images show mostly domains that are agglomerated and not clearly separated primary nanoparticles. The constituent primary particles have overlapping boundaries, and cannot be reliably distinguished as a function of the available SEM images for this, the primary-particle diameter has not been determined. However, these apparent lateral size dimensions of well-defined agglomerates were recorded to give a semi-quantitative characterization of the morphological size distribution. The measurements should thus be regarded as an agglomerate size and not as a primary particle size.

##### SEM-derived agglomerate-size distribution

**Fig. 7:**
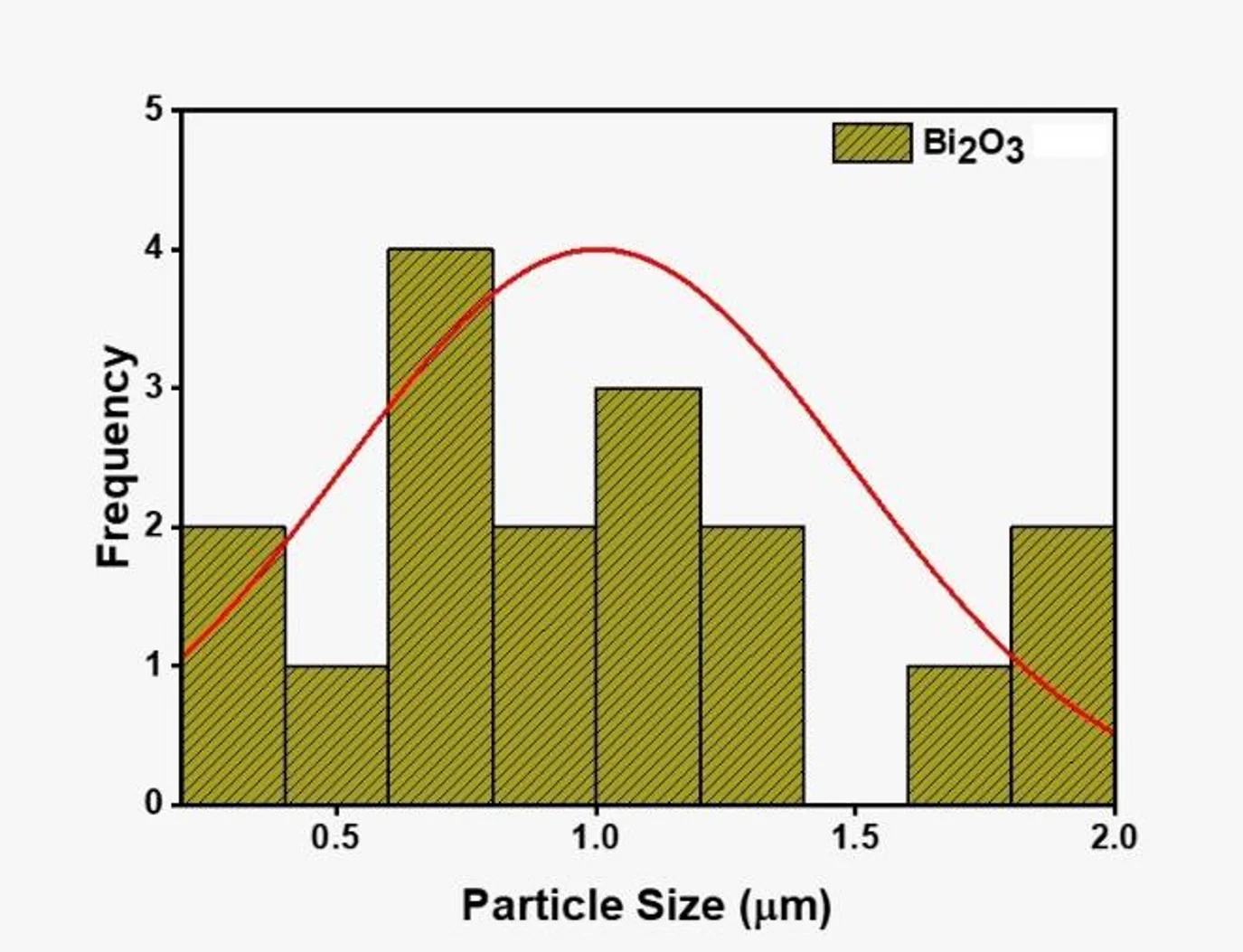
Apparent diameter frequency distribution of the SEM visible agglomerates of the sample of biosynthesized Bi₂O₃. Images were captured from a calibrated SEM micrograph and measurements were taken from these. The red curve is the best fit distribution and is shown as an aid to the observed size trend. The reported dimensions are for agglomerated domains and not for primary nanoparticle diameters

The apparent lateral size of well-defined agglomerates was determined by measuring from calibrated SEM micrographs to complement the qualitative SEM observations. The measured agglomerate sizes were in the range of 0.20–2.00 μm as depicted in Fig. 7. Very little of the material observed by SEM was in the micrometre range, the most numerous size interval being 0.60–0.80 μm. This distribution was rather wide and other agglomerates reached into the micrometre-size domain.

The fitted distribution had a peak at ~ 1.00 μm. This number, however, is the size of agglomerated domains which can be seen in SEM, not the actual diameter of primary nanoparticles. The wide spread is in line with the irregular and heterogeneous aggregation seen in the SEM micrographs. This type of aggregation can be caused by interactions between the growing Bi₂O₃ crystallites and the surface constituents of plants during synthesis and drying, but the present SEM analysis cannot directly prove any particular aggregation mechanism.

Appparent agglomerate size from SEM is bigger than the crystallite size from XRD. This distinction is scientifically justified as the latter is measuring the size of crystallites with coherent diffraction and the former is measuring larger surface structures which can include several crystallites. Thus, the agglomerate size (obtained from SEM) and XRD crystallite size refer to distinct structural levels of the synthesized material and are therefore not interpreted as conflicting.

### Antimicrobial evaluation of Bi_2_O_3_ NPs

The antimicrobial activity of the biosynthesized Bi_2_O_3_ NPs was tested on two bacterial strains, *Staphylococcus aureus* and *Escherichia coli*, and two fungal strains, *Candida albicans* and *Aspergillus niger*. The agar well diffusion technique and minimum inhibitory concentration values were used to measure the antibacterial and antifungal activities [28, 29].

The findings revealed that there was an increase in the inhibition zones with the concentration against both bacterial strains. In the case of *S. aureus*, the inhibition zone expanded to 24 mm at 30 µg/mL as compared to 13 mm at 2 µg/mL. In the case of *E. coli*, the inhibition zone expanded to 10 mm with 2 µg/mL to 25 mm with 20 µg/mL (Figure 9). *E. coli* and *S. aureus* had MIC values of 15 µg/mL and 30 µg/mL, respectively, indicating that *E. coli* was more sensitive to the Bi₂O_3_ NPs that were synthesized compared to *S. aureus*. This distinction can be attributed to the differences in bacterial cell wall organization and membrane permeability of Gram-negative and Gram-positive bacteria.

The Bi_2_O_3_ NPs synthesized were also shown to have antifungal properties against *Candida albicans* as well as *Aspergillus niger*. Inhibition zone of *Candida albicans* was 20 mm at 10 µg/mL and 32 mm at 30 µg/mL, but *Aspergillus niger* gave an inhibition zone of 16 mm to 27 mm at the same range of concentration. MIC of *Candida albicans* was 15 µg/mL, whereas *Aspergillus niger* had 30 µg/mL, which showed increased sensitivity of *Candida albicans* to the produced nanoparticles [30, 31].

**Fig. 8:**
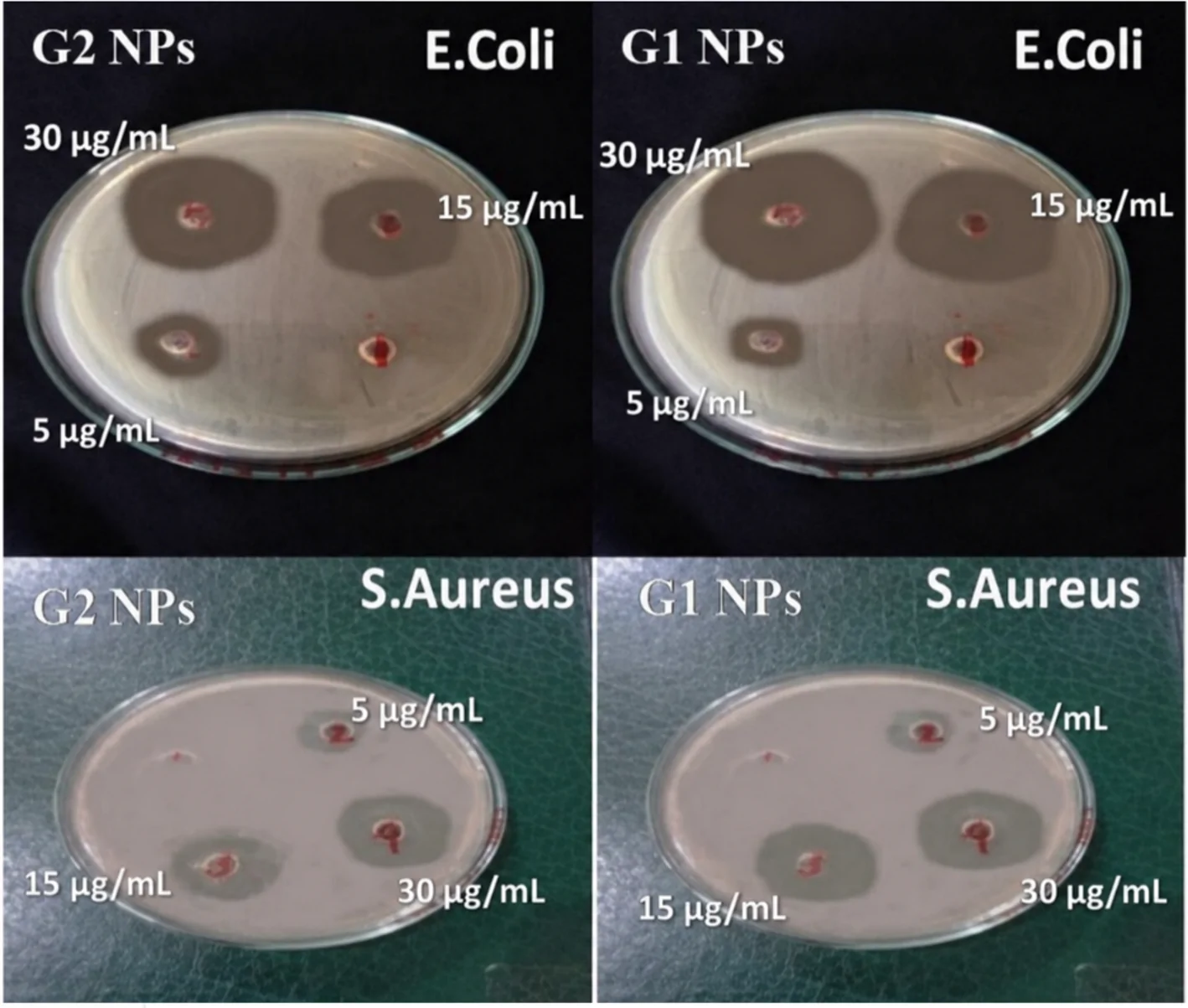
Antibacterial activity of biosynthesized Bi_2_O_3_ NPs against *Escherichia coli* and *Staphylococcus aureus* evaluated by the well diffusion method

Bi_2_O_3_ NPs antimicrobial activity can be explained by the potential interaction with microbial cell envelopes, the potential formation of oxidative stress, and disruption of normal cellular functions. Moreover, the nanoparticle surface has plant-derived phytochemicals, which could have been the source of the observed biological activity, which enhances contact between the nanoparticles and the microbial cells. Porous and aggregated morphology of SEM could also offer several sites of interaction at the surface. Nevertheless, the exact mechanism of action of antimicrobials should be validated by additional mechanistic research, including ROS detection, membrane integrity testing, and observation of treated microbial Fig. 8 cells under the microscope [32, 33].

In general, the findings indicate that *Cuminum cyminum* mediated Bi_2_O_3_ NPs have concentration-dependent antibacterial and antifungal properties, and better activity against *E. coli*, and *Candida albicans*. These results indicate that the prepared Bi_2_O_3_ NPs can potentially be used as antimicrobial nanoparticles [34].

**Fig. 9:**
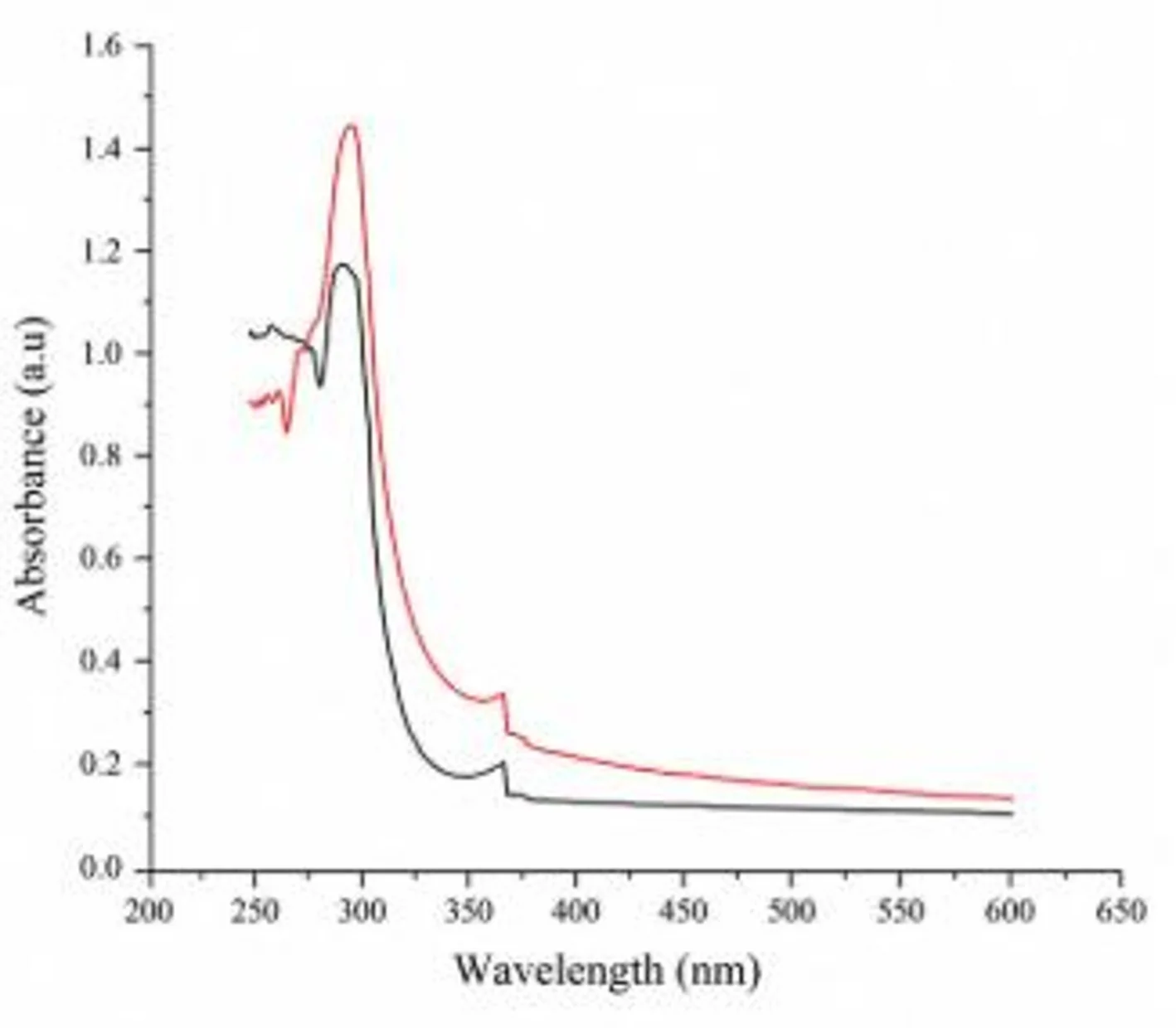
Antifungal activity of biosynthesized Bi_2_O_3_ NPs against *Candida albicans* and *Aspergillus niger* evaluated using the well diffusion method

Figure 9; Tables 2 and 3 also show the effect of concentration dependence of the antifungal activity. *Candida albicans* were more sensitive and the inhibition zones were between 20 mm at 10 ug/mL up to 32 mm at 30 ug/mL. In *Aspergillus niger* the range of concentrations between 16 mm and 27 mm was inhibited. The MIC values also prove that *C. albicans* is more vulnerable to Bi_2_O_3_ NPs (MIC = 15 µg/mL) than *A. niger* (MIC = 30 µg/mL), which is probably due to the difference in the cell wall structure and membrane permeability [35, 36].

**Table 2:** Zone of inhibition of Bismuth oxide nanoparticles (Bi_2_O_3_ NPs) against fungal strains

Order	Fungus	Concentration on (µg/mL)	Inhibition-zone diameter (mm), mean ± SD
1	*Candida albicans*	10	20
2	*Candida albicans*	15	26
3	*Candida albicans*	30	32
4	*Aspergillus niger*	10	16
5	*Aspergillus niger*	15	21
6	*Aspergillus niger*	30	27

**Table 3:** Minimum inhibitory concentrations (MIC, µg/mL) of bismuth nanoparticles (BiNPs) against fungal strains

Order	Fungus	MIC (µg/mL)
1	Candida albicans	15
2	Aspergillus niger	30

The fact that the concentration-dependent size of the inhibition zone of both bacteria and fungi are increased suggests that Bi_2_O_3_ NPs can be used to effectively disrupt the microbial cell membrane and affect the most vital processes in cells [37]. The increased sensitivity of *E. coli* and *C. albicans* can be explained [38] by the fact that the cells with a relatively thin cell wall or more permeable membrane can be penetrated by nanoparticles better. This data confirms that bismuth oxide nanoparticles prepared using cumin extract are efficient antimicrobial agents and can be utilized in the future in the treatment of bacterial and fungal infections. Further research needs to clarify the exact action of the Bi_2_O_3_ NPs and the optimal concentration of the Bi_2_O_3_ NPs to be used in the clinical and environmental settings [39].

### Cytotoxicity assessment of bismuth oxide nanoparticles

Cytotoxic effect of Bismuth oxide nanoparticles (Bi_2_O_3_ NPs) was tested on HeLa and MCF-7 human cancer cell lines through the MTT assay [23, 40]. The IC_50_ values showed a time-dependent cytotoxicity rise. In the case of HeLa cells, the IC_50_ decreased with time, 25 µg/mL at 24 h to 15 µg /mL at 72 h, which suggests that cytotoxicity increased with exposure time. Equally, at 24 h, IC_50_ of MCF-7 cells was 35 ug/ml and IC_50_ of 72 h was 25 ug/ml, an increase in cytotoxicity by moderately but significantly significant (Table 4).

**Table 4:** Half-maximal inhibitory concentration (IC₅₀, µg/mL) of bismuth nanoparticles (BiNPs) against HeLa and MCF-7 cancer cell lines

Order	Cell Line	IC50 (µg/mL)	24 h	48 h	72 h
1	HeLa	20	25	20	15
2	MCF-7	30	35	30	25

These were also demonstrated by cell viability analysis. In HeLa cells, treatment with 10 µg/mL Bi_2_O_3_ NPs resulted in 85% viability at 24 h, which declined to 70% at 72 h. At the highest tested concentration of 50 µg/mL, viability dropped sharply to 10% at 72 h. In MCF-7 cells, 10 µg/mL Bi_2_O_3_ NPs led to 90% viability at 24 h, decreasing to 75% at 72 h, while 50 µg/mL reduced viability to 20% at 72 h (Table 5). These results indicate a clear dose- and time-dependent cytotoxic effect of Bi_2_O_3_ NPs on both cancer cell lines.

**Table 5:** Cell viability (%) of HeLa and MCF-7 cancer cell lines treated with varying concentrations of bismuth oxide nanoparticles (Bi2O3 NPs)

Order	Cell Line	Concentration (µg/mL)	24 hours	48 hours	72 hours
1	HeLa	10	85	80	70
2	HeLa	25	60	50	40
3	HeLa	50	35	20	10
4	MCF-7	10	90	85	75
5	MCF-7	25	70	60	50
6	MCF-7	50	40	30	20

The results demonstrate that Bi_2_O_3_ NPs effectively inhibit cancer cell proliferation in a dose- and time-dependent manner. The more pronounced cytotoxicity in HeLa cells compared to MCF-7 cells may be due to differences in membrane permeability, metabolic activity, or intrinsic cellular sensitivity to nanoparticles.

The presence of several mechanisms that bismuth oxide nanoparticles are likely to cause their cytotoxic effects includes the production of reactive oxygen species (ROS), mitochondrial dysfunction, disruption of DNA replication and mitosis blockage. The increased vulnerability of the HeLa cells implies that there will be an increased contact between Bi_2_O_3_ NPs and the cellular components resulting in increased apoptotic or necrotic cell death. In general, these results demonstrate the possibility of Bi_2_O_3_ NPs in the treatment of cancer through targeted therapy.

### Performance evaluation of Bi_2_O_3_ NPs

The present study exhibits better performance criteria of Bismuth oxide nanoparticles (Bi_2_O_3_ NPs) synthesized by cumin seed extract as compared to the studies reported in the past (Table 6). The synthesized material exhibited crystalline Bi₂O₃ features with possible contributions from monoclinic α-Bi₂O₃ and tetragonal β-Bi₂O₃, together with minor unidentified reflections. SEM revealed irregular, rough and strongly agglomerated structures with a broad apparent agglomerate-size distribution. These Bi_2_O_3_ NPs were broad-spectrum antimicrobial agents, and they were highly sensitive to both bacterial (*Escherichia coli* and *Staphylococcus aureus*) and fungal (*Candida albicans* and *Aspergillus niger*) strains (Tables 2, 3, 7 and 8). This shows that the present nanoparticles are more efficient than those mentioned by Vazquez-Munoz et al. [41] and other researchers because the latter mostly concentrated on restricted strains or lacked fungal pathogens. Also, cytotoxicity assay showed a decrease in 72 h IC_50_ against HeLa (15 ug/mL) and MCF-7 (25 ug/mL) cancer cell lines (Tables 4 and 5), indicating superior anticancer potential over the already reported Bi_2_O_3_ NPs, including those investigated by Akhtar, M. J. et al. [42] and Shakibaie, Mojtaba, et al. [43], with higher IC_50_ values or no cytotoxic information All in all, a mixture of green synthesis, increased antimicrobial activity, and a high cytotoxic potential makes the present study offer the synthesized Bi₂O₃ nanoparticle with high biomedical and environmental applicability.

**Table 6:** Comparative analysis of bismuth nanoparticles (Bi2O3 NPs)

Order	Study	Synthesis Method	Antimicrobial Activity	Cytotoxicity (IC₅₀, µg/mL)	Key Findings	Ref.
1	Current Study	Green synthesis using cumin seed extract	Effective against E. coli, S. aureus, C. albicans, A. niger	HeLa: 15 (72 h), MCF-7: 25 (72 h)	Strong antimicrobial and cytotoxic effects with eco-friendly synthesis	This study
2	Plant-mediated BiNPs	Green synthesis (using plant extracts)	Active against S. aureus and C. albicans	Not specified	Demonstrated broad-spectrum antimicrobial activity	[41]
3	Bi₂O₃ Nanoparticles (Chemical)	Chemical synthesis	Not specified	HUVE cells: 6.7 (24 h)	Selective cytotoxicity in human endothelial cells	[42]
4	Biologically synthesized BiNPs	Biological synthesis	Not specified	HT-29 cells: 28.7	Cytotoxic effect on colon cancer cells	[43]

**Table 7:** Zone of inhibition (mm) of Bismuth oxide nanoparticles (Bi2O3 NPs) against different bacterial strains

Order	Bacteria	Concentration (µg/mL)	Inhibition-zone diameter (mm), mean ± SD
1	*S. aureus*	0.00	0
2	*S. aureus*	2	13
3	*S. aureus*	5	15
4	*S. aureus*	15	19
5	*S. aureus*	30	24
6	*E. coli*	0.00	0
7	*E. coli*	2	10
8	*E. coli*	5	12
9	*E. coli*	15	21
10	*E. coli*	20	25
11	*E. coli*	30	29

**Table 8:** Minimum inhibitory concentrations (MIC, µg/mL) of bismuth nanoparticles (BiNPs) against bacterial strains

Order	Bacteria	MIC (µg/mL)
1	E. coli	15
2	S. aureus	30

## Conclusion

This paper was able to show that Bismuth oxide nanoparticles (Bi_2_O_3_ NPs) can be synthesised greenly through the use of cumin seed extract as a natural reducing and stabilizing agent to bismuth nitrate. XRD indicated crystalline Bi₂O₃ with possible mixed α- and β-phase contributions, while SEM revealed irregular and strongly agglomerated structures. The apparent dimensions of clearly distinguishable agglomerates extended from approximately 0.20 to 2.00 μm, with the highest frequency in the 0.60–0.80 μm interval. However, the diameter of individual primary nanoparticles could not be determined reliably from the available SEM images. The formation, structural integrity, and stabilization of Bi_2_O_3_ NPs was successfully achieved and confirmed using extensive characterization of UV-Visible spectroscopy, FTIR, X-ray diffraction (XRD), scanning electron microscopy (SEM) analysis, in addition to uncovering the unique optical and morphological characteristics of the product. The resulting Bi_2_O_3_ NPs exhibited significant antimicrobial behavior towards bacterial and fungal cultures with *Candida albicans* being the most sensitive microorganism to the Bi_2_O_3_ NPs. Moreover, the nanoparticles were found to be highly cytotoxic to the HeLa and MCF-7 cell lines of human cancer cells lines, supported their utility as therapeutic agents. Such findings indicate the effectiveness of cumin seed extract in the synthesis of environmentally friendly nanoparticles and are indicative of the potential of Bi_2_O_3_ NPs in biomedical applications, such as the use of antimicrobial therapies and cancer treatment, and environmental technologies.

## Data Availability

All data generated or analyzed during this study are included in this article.
